# Editorial: Drug-induced immunogenic cell death patterns and anti-cancer treatment

**DOI:** 10.3389/fphar.2023.1252168

**Published:** 2023-07-13

**Authors:** Xianghou Xia, Zu Ye, Sajjad Ahmad, Yongkun Wei, Yang Yu

**Affiliations:** ^1^ Department of Breast Surgery, Institute of Basic Medicine and Cancer (IBMC), Chinese Academy of Sciences, The Cancer Hospital of the University of Chinese Academy of Sciences (Zhejiang Cancer Hospital), Hangzhou, China; ^2^ Zhejiang Cancer Hospital, University of Chinese Academy of Sciences, Hangzhou, China; ^3^ Department of Health and Biological Sciences, Abasyn University, Peshawar, Pakistan; ^4^ Department of Molecular and Cellular Oncology, University of Texas MD Anderson Cancer Center, Houston, TX, United States

**Keywords:** immunogenic cell death, cancer, anoikis, apoptosis, pyroptosis, ferroptosis

## Introduction

Immunogenic cell death (ICD) induced by certain drugs has emerged as a promising avenue in cancer treatment. Understanding the underlying mechanisms and identifying key factors involved in drug-induced ICD can pave the way for innovative therapeutic approaches. In this editorial, we discuss the findings from five recent articles that shed light on drug-induced ICD patterns and their implications for anti-cancer treatment. These studies explore various aspects, including prognostic risk models, therapeutic targets, immunotherapy response, and molecular subtypes, providing valuable insights for personalized medicine and precision therapy in different types of cancer.

## Unraveling drug-induced immunogenic cell death patterns

Zhou et al. presented a novel risk model based on anoikis-related genes (ARGs) that accurately predicts prognosis and immune infiltration in cutaneous melanoma. Their study demonstrates the potential of combining targeted therapy and immunotherapy for improved treatment outcomes in this aggressive skin cancer.

Wang et al. investigated the role of autophagy-related gene LAPTM4B in renal clear cell carcinoma (RCC). Their findings highlight the association between LAPTM4B expression and the tumor immune microenvironment, suggesting LAPTM4B as a potential immunotherapeutic target for RCC.

Li et al. constructed a prognostic risk model based on anoikis-related long non-coding RNAs (lncRNAs) in gastric adenocarcinoma (STAD). Their research provides a deeper understanding of STAD carcinogenesis and offers a prognostic tool for precise therapy in gastric cancer patients.

Zhai et al. provided an update review on chemotherapeutic and targeted drugs-induced ICD in cancer models. They emphasize the molecular mechanisms underlying ICD and discuss the potential of ICD in cancer immunotherapy, highlighting the prospects for chemoimmunotherapy development.

Hu et al. explored lysosome-associated genes (LYAGs) and their role in gastric cancer (GC). They identified molecular subtypes based on LYAGs and establish a prognostic risk signature, providing insights into clinical prognosis and immune infiltration in GC.

## Implications for anti-cancer treatment and personalized medicine

The findings from these studies collectively contribute to the growing body of evidence supporting the integration of drug-induced ICD patterns into anti-cancer treatment strategies. Drug-induced ICD holds immense potential in enhancing the immune response against tumors, thereby improving patient outcomes and expanding the repertoire of treatment options.

Personalized medicine is a recurring theme across these studies. By identifying prognostic risk models, therapeutic targets, and molecular subtypes, clinicians can tailor treatment approaches to individual patients, maximizing efficacy while minimizing adverse effects. The ability to predict prognosis, assess immunotherapy response, and determine drug sensitivity based on specific ICD patterns opens up new avenues for precision therapy.

## Challenges and future directions

While the potential of drug-induced ICD in cancer treatment is promising, several challenges lie ahead. Developing reliable biomarkers for predicting ICD response and understanding mechanisms of resistance to ICD induction are crucial for optimizing clinical practice and designing effective therapeutic strategies.

Further collaborative efforts among researchers, clinicians, and pharmaceutical industries are essential for translating these scientific discoveries into clinical applications. By fostering interdisciplinary collaborations, we can expedite the development of innovative treatment regimens and facilitate the integration of drug-induced ICD patterns into routine clinical practice.

## Conclusion

The recent research on drug-induced immunogenic cell death patterns provides compelling evidence of its potential in anti-cancer treatment. The identified risk models, therapeutic targets, and molecular subtypes offer valuable insights for personalized medicine and precision therapy. As we continue to unravel the complexities of drug-induced ICD, we move closer to a future where tailored treatment approaches based on individual ICD patterns become a reality. Together, we can revolutionize cancer treatment and bring about a new era of hope and healing.

